# Evaluating Cognitive Bias in Psychosis: A Novel Approach Using Large Language Models on Spanish Speech Data

**DOI:** 10.1192/j.eurpsy.2025.771

**Published:** 2025-08-26

**Authors:** E. Gutierrez Alvarez, A. Barajas, R. Ayesa

**Affiliations:** 1 Universidad Politécnica de Madrid, NEBULA group, Madrid, Spain; 2 MIT linQ - Massachusetts Institute of Technology, Cambridge, United States; 3 Department of Clinical and Health Psychology, Autonomous University of Barcelona; 4 Government of Catalonia, Serra Húnter Programme, Barcelona; 5 Mental Illness Research Group, Valdecilla Biomedical Research Institute (IDIVAL), Santander; 6 Biomedical Research Center in Mental Health Network (CIBERSAM), Health Institute Carlos III, Madrid, Spain

## Abstract

**Introduction:**

Jumping to conclusions (JTC) is a cognitive bias strongly involved in the genesis of psychotic symptoms. Accurate evaluation of JTC is crucial for early intervention and treatment planning. However, traditional assessment methods are time-consuming and subject to human error. This study leverages state-of-the-art Large Language Models (LLMs) to evaluate JTC in a unique Spanish population database collected through the DISCOURSE protocol at the Instituto de Investigación Marqués de Valdecilla (IDIVAL).

**Objectives:**

Our primary objectives were to:

1. Assess the efficacy of LLMs in evaluating JTC bias from transcribed speech.

2. Compare different LLM models and prompting techniques for optimal performance.

3. Explore the potential of AI-assisted cognitive bias evaluation in clinical settings.

**Methods:**

We utilized a database of approximately 170 participants, including patients, controls, and relatives, collected through the DISCOURSE protocol. This protocol is particularly valuable as it includes tasks designed to elicit JTC behaviors, such as ambiguous picture interpretation. Audio recordings were automatically transcribed using two speech-to-text algorithms and manually revised for accuracy.

We investigated various LLM models (“gpt4o”, “claude-sonnet-3.5”, “llama3”, “gemini pro”) and experimented with different prompting techniques, including instruction combinations and reasoning scratchpads (Chain of Thoughts).

**Results:**

Our evaluation has provided valuable insights into the potential of LLMs for assessing Jumping to Conclusions (JTC) bias. We observed varying degrees of effectiveness across different LLM models in identifying JTC behaviors from transcribed speech, with some showing promise in capturing subtle linguistic cues. Prompting techniques, particularly Chain of Thought reasoning, demonstrated potential in enhancing the models’ analytical capabilities. Given our Spanish-language database, we gained important insights into LLM performance in non-English contexts. Error analysis identified common limitations, informing future refinements. Preliminary findings suggested performance variations across demographic subgroups, highlighting areas for further investigation.

**Image 1:**

**Figure:**
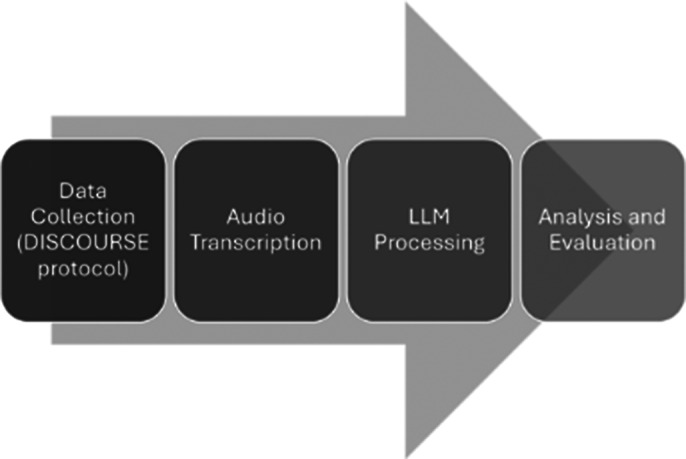

**Conclusions:**

This study represents a step towards integrating AI and automation into clinical workflows for psychosis evaluation and treatment. The understanding of the ability of LLMs to assess JTC from speech samples could significantly enhance the objectivity of cognitive bias evaluations. These findings lay the groundwork for future research exploring the integration of AI in psychosocial interventions for psychosis, including potential applications in cognitive remediation, metacognitive training, and personalized treatment planning.

**Disclosure of Interest:**

None Declared

